# Guillain-Barre syndrome observed with adoptive transfer of lymphocytes genetically engineered with an NY-ESO-1 reactive T-cell receptor

**DOI:** 10.1186/s40425-019-0759-x

**Published:** 2019-11-08

**Authors:** Jocelyn Joseph, Michael J. Nathenson, Van Anh Trinh, Karan Malik, Erica Nowell, Kristen Carter, Shiao-Pei Weathers, George D. Demetri, Dejka Araujo, Anthony P. Conley

**Affiliations:** 1 Department of Clinical Pharmacy Services, The University of Texas MD Anderson Cancer Center, 1515 Holcombe Blvd, Houston, TX USA; 2 Center for Sarcoma and Bone Oncology, Dana-Farber Cancer Institute, 450 Brookline Ave, Brighton, MA USA; 3 Department of Sarcoma Medical Oncology, The University of Texas MD Anderson Cancer Center, 1515 Holcombe Blvd, Houston, TX USA; 4 Department of Neuro-Oncology, The University of Texas MD Anderson Cancer Center, 1515 Holcombe Blvd, Houston, TX USA

**Keywords:** Guillain-Barre syndrome, Synovial sarcoma, Adoptive T-cell transfer, NY-ESO-1

## Abstract

**Background:**

Adoptive transfer of autologous T-lymphocytes transduced with a high affinity NY-ESO-1-reactive T-cell receptor (NY-ESO-1^c259^ T-cells) has emerged as a promising therapeutic strategy for patients with refractory synovial sarcoma. Secondary autoimmune T-cell mediated toxicities can occur long after initial adoptive T-cell transfer. We report on the first two cases of the development and management of Guillain-Barre syndrome in synovial sarcoma patients who received NY-ESO-1^c259^ T-cells.

**Case presentation:**

A 47 year-old woman and 39 year-old woman with refractory metastatic SS were treated with fludarabine-cyclophosphamide lymphodepletion followed by adoptive transfer of NY-ESO-1^c259^ T-cells. On day 42 after adoptive T-cell therapy, patient one presented to the emergency room with a one-week history of numbness, paresthesia, and heaviness to both legs progressing to difficulty walking on the day of presentation. Although MRI brain and lumbar puncture were negative, electromyography (EMG) and nerve conduction studies (NCS) of the lower extremities and right arm performed revealed an abnormal study suggestive of a very mild, distal, motor, axonal polyneuropathy. Patient two presented on day 113 with bilateral foot numbness, left foot drop, unsteady gait, and pain in the left thigh, which progressed over two week to bilateral leg weakness, inability to walk, and numbness bilaterally in the hands, legs, and feet. Both patients received intravenous immunoglobulin (IVIG) 0.4 g/kg/day for 5 days for possible acute inflammatory demyelinating polyneuropathy (AIDP) likely related to NY-ESO-1 targeting T-cell therapy. After 3 and 5 doses, respectively, of IVIG, the patients reported improvement in symptoms and strength, and were later transferred to an inpatient rehabilitation facility to continue gaining strength. At patient one’s neurology follow-up on day 95, she reported only mild left lower extremity (LLE) weakness and was gradually successfully regaining independence in motor function. At patient two’s 9-month follow-up, the patient had regained normal function and independence.

**Conclusions:**

Given the expanding applications of immunotherapy in cancer management, clinicians should stay vigilant against the potential development of unusual but life-threatening immune-mediated toxicities.

## Background

Synovial Sarcoma (SS) accounts for approximately 6–10% of all soft tissue sarcomas (STS). SS mostly affects young adults, with the peak incidence in the third decade of life [1]. Molecularly, it is characterized by the translocation t(X;18)(p11;q11), involving the *SYT* gene at 18q11 and the *SSX1*, *SSX2*, or *SSX4* gene at Xp11 [2, 3]. SSs are aggressive STS with a high propensity to metastasize. Despite the current standard-of-care chemotherapy, recurrent and metastatic SSs are almost always fatal, with a median time to cancer–specific death of 10–22 months [4, 5]. There is an urgent need for new and effective treatments.

Although checkpoint inhibitors have gained a firm foothold in the management of many solid tumors, they are ineffective in treating SS [6–8]. A phase II study evaluating ipilimumab, a cytotoxic T-lymphocyte antigen-4 (CTLA-4) inhibitor, in patients with advanced SS was terminated after rapid disease progression was noted in the first 6 patients [6]. The clinical activity of blocking antibodies targeting programmed cell death-1 (PD-1) in SS was not encouraging. Only one of 10 synovial sarcoma patients achieved a short-lived partial response in SARC028, a phase II study evaluating pembrolizumab, an anti-PD-1 antibody, in patients with advanced STS and bone sarcomas [7]. Even the combination of nivolumab and ipilimumab, which has demonstrated synergistic activity against other solid tumors, did not fare any better in terms of treatment outcomes in SS. In the Alliance A091401, a phase II study evaluating nivolumab monotherapy and ipilimumab-nivolumab in two separate non-comparative randomized cohorts, 6 out of 38 evaluable patients with advanced STS responded to the combination. Unfortunately, none of the responders had advanced SS [8].

NY-ESO-1 (New York esophageal squamous cell carcinoma 1) is a cancer-testis antigen that is expressed at high levels in 70–80% of SS cases [9, 10]. A peptide epitope corresponding to amino acids 157 to 165 of NY-ESO-1 can be recognized by HLA-A2-restricted CD8+ T-cells [11]. Adoptive transfer of autologous T lymphocytes transduced with a high affinity NY-ESO-1-reactive T-cell receptor (NY-ESO-1^c259^ T cells) has emerged as a promising therapeutic strategy for patients with refractory SS. Results of the pilot study of NY-ESO-1^c259^ T cells followed by high-dose interleukin-2 (NCT00670748) by Robbins et al. indicated that 11 of 18 (61%) heavily pretreated patients with NY-ESO-1-expressing SS achieved objective tumor responses, with the estimated 3- and 5-year overall survival rates of 38 and 14%, respectively [12]. More recently, an overall response rate of 50% was demonstrated with NY-ESO-1^c259^ T cells without high-dose interleukin-2 in the first cohort of 12 previously-treated patients with metastatic SS in an ongoing phase I/II study (NCT01343043) [13]. Many other clinical trials evaluating NY-ESO-1 targeting T-cell therapies in advanced SS are underway.

In this case series, we discuss the presentation and management of Guillain-Barre syndrome in two patients treated with lymphodepletion and subsequent NY-ESO-1^c259^ T-cells, on Adaptimmune protocol ADP-04511 (Table 1).

## Case presentation #1

A 47 year-old female with refractory metastatic SS was treated with fludarabine-cyclophosphamide lymphodepletion (Table 1) followed by adoptive transfer of NY-ESO-1^c259^ T-cells. Prior oncologic treatments included radiotherapy followed by surgical resection of a 9.4 cm right paraspinal mass with negative margins. Upon presentation to our institution, she was found to have metastases involving the right inguinal lymph nodes, lungs, and T-spine, for which she received ifosfamide for 4 cycles followed by doxorubicin monotherapy for 6 cycles, with positive response. Upon progression, she received pazopanib and palliative radiation to osseous metastases in the thoracic and lumbar spines. She was then enrolled in the Adaptimmune ADP-04511 protocol. Her tumor had had low NY-ESO-1 expression, defined as ≥1+ by IHC in ≥1% cells but not exceeding 2+ or 3+ in ≥50% cells.

**Table 1:** Summary of Past Oncologic History & Management of GBS/AIDP

	Patient 1 47-year old female	Patient 2 39-year old female
Primary tumor	9.4 cm right paraspinal mass	8 cm left thigh mass
Prior chemotherapy	• Ifosfamide ×4 cycles • Doxorubicin ×6 cycles • Treatment break/surveillance • Pazopanib	• Doxorubicin/ifosfamide × 4 cycles • Pazopanib
Sites of disease prior to lymphodepletion	• Bilateral lungs, spine, right groin	• Lung, local thigh recurrence
Lymphodepletion regimen	• Fludarabine 20 mg/m2 (dose reduced from 30 mg/m2 due to renal dysfunction per protocol) daily ×4 days • Cyclophosphamide 1800 mg/m2 daily × 2 days	• Fludarabine 30 mg/m2 daily × 4 days • Cyclophosphamide 1800 mg/m2 daily × 2 days
Onset of Symptoms of GBS/AIDP	• Day 42: 1-week history of numbness, paresthesia, heaviness to both legs; difficulty walking on day 42; pt. declined admission	• Month 4 follow-up visit: bilateral foot numbness, left foot drop, unsteady gait, and pain in left thigh
Admission for workup of symptoms	• Day 46: admitted for workup; numbness & paresthesias w/hypoesthesia starting with feet & ascending to hips bilaterally	• Day 128: admitted for workup; additional worsening neurologic symptoms of peripheral sensory and motor neuropathy
Electromyography / nerve conduction studies	• Day 48: very mild, distal motor, axonal polyneuropathy	• Non-length dependent demyelinating sensorimotor polyneuropathy
Lumbar Puncture	• Day 49: CSF with no pleiocytosis, malignant cells, infectious processes, or albuminocytologic dissociation	• CSF with no malignant cells, low cell count, no bacteria, negative viral studies
Intravenous Immuneglobulin (IVIG)	• Day 48–52: IVIG 0.4 g/kg/day for 5 days	• IVIG 0.4 g/m/day for 5 days
Improvement of Symptoms	• Day 50: improvement in symptoms & strength per patient	• Improved strength the day after completion of IVIG
Able to Ambulate	• Day 60: with walker under supervision	• 6-month follow-up visit: strength and sensory neuropathy continued to improve, but still using walker

She tolerated lymphodepletion and T-cell infusion well, with grade 1 cytokine release syndrome (CRS) manifesting as almost daily fevers > 38.3 °C, with associated chills and tachycardia, until Day 6. She was noted to have an elevated C-reactive protein (CRP) and elevated serum ferritin. Empiric antibiotics were initiated, but no infectious organisms were identified by serial cultures. The patient developed a diffuse, macular, blanching rash on day 10 that was biopsied and found to be a drug rash (sparse perivascular lymphohistiocytic infiltrate, rare dyskeratotic keratinocytes, pigment incontinence), which improved when levetiracetam and meropenem were discontinued. She developed grade 1 neurotoxicity (mild confusion), which fully resolved on day 18. She was discharged on day 28 due to delayed neutrophil recovery. Restaging evaluation on day 32 demonstrated interval reduction in size and number of lung and nodal metastases.

On day 42, she presented to the emergency room with a one-week history of numbness, paresthesia, and heaviness to both legs progressing to difficulty walking on the day of presentation. Physical exam revealed loss of strength to the iliopsoas groups and absent deep tendon reflexes (DTRs) in bilateral lower extremities (BLEs). MRI-C/T/L spine revealed no evidence of cord compression or transverse myelitis. Patient declined admission; therefore, she was discharged with a neurology consultation scheduled on day 46.

On the day of the neurology consultation, she reported numbness and paresthesias with associated hypoesthesia that started with her feet and ascended to her hips bilaterally, progressing to leg weakness first involving the distal aspect of her legs, which were more affected at that time than her proximal legs. She denied bowel/bladder dysfunction, dysphagia, dyspnea, or back pain. Vital signs were remarkable for sinus tachycardia. Patient was fully alert and oriented. The cranial nerve exam was unremarkable. The motor exam was notable for bilateral bicep and lower extremity weakness involving the non-antigravity muscles to a greater extent than the antigravity muscles. Mild pseudoathetosis was noted on legs. DTRs were markedly diminished in BLEs. The sensory exam revealed stocking distribution in the decremental response distally with reduced sensation to all modalities (light touch, pinprick, vibration, temperature, proprioception). Patient required assistance to stand with an ataxic gait. The patient was admitted for further workup.

An MRI-brain performed on day 47 was unremarkable. As the majority of cases of GBS are caused by the immune response following a preceding infection, infectious workup was conducted. Comprehensive infectious disease workup was negative (including Coxiella-Rickettsia, Lyme disease, Zika, CMV, EBV). Of note, camphylobacter jejuni was not tested for via GI multiplex as the patient did not develop diarrhea. Lumbar puncture (LP) was performed on day 49, and cerebrospinal fluid (CSF) revealed no pleiocytosis, malignant cells, infectious processes (including HHV6, HSV1/2, VZV, CMV, Enterovirus, West Nile), or albuminocytologic dissociation. Of note, LP was performed after IVIG therapy was initiated. Regarding the patient’s autoimmune laboratory workup, oligoclonal bands were identified in serum but not in CSF protein immunofixation electrophoresis. CRP and erythrocyte sedimentation rate (ESR) was elevated at 44.9 mg/dL and 30 mm/hr. respectively. All other autoimmune laboratory workup, including anti-nuclear panel, paraneoplastic panel, acetylcholine receptor binding antibody, ganglioside antibody panel were negative. Electromyography (EMG) and nerve conduction studies (NCS) of the BLEs and right arm performed on day 48 revealed normal and symmetric brisk responses, except borderline slowed conduction velocity in the distal segment on the left recording extensor digitalis brevis. The consulting neurologist’s impression was that the electrophysical findings were suggestive of a very mild, distal, motor, axonal polyneuropathy.

The patient received intravenous immunoglobulin (IVIG) 0.4 g/kg/day for 5 days starting on day 48 for a working diagnosis of acute inflammatory demyelinating polyneuropathy (AIDP) likely related to NY-ESO-1 targeting T-cell therapy. After 3 doses of IVIG, the patient reported improvement in symptoms and strength. By day 60, the patient was able to ambulate with a walker under supervision. The patient was transferred to an inpatient rehabilitation medicine service. During the patient’s neurology follow-up on day 95, she reported only mild left lower extremity weakness, that she was continuing to work with physical therapy, and was largely independent; thus, the neurology team decided she did not require another course of IVIG.

**Fig. 1:**
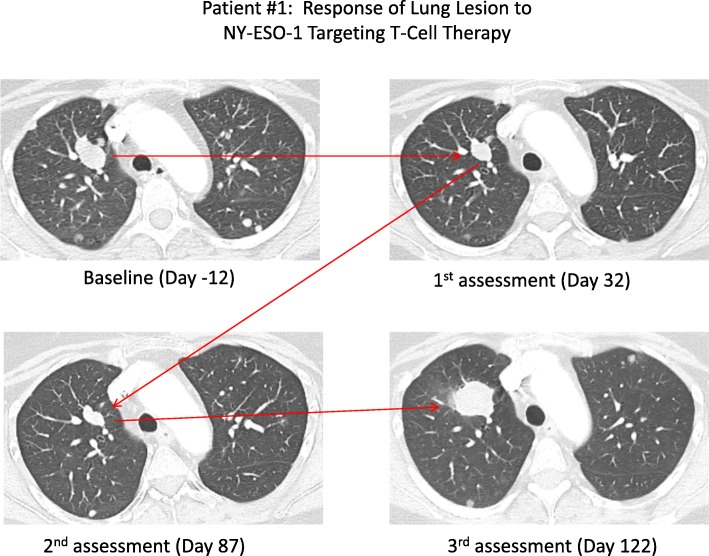
Patient #1: Response of lung lesion to NY-ESO-1 targeting T-Cell therapy

With regard to the patient’s synovial sarcoma, day 87 (week 12) restaging scans revealed stable disease, but unfortunately, day 122 (week 17) restaging scans revealed progression of pulmonary metastases (Fig. 1), enlarging soft-tissue mass near lumbar spine, and stable thoracic and lumbar spinal metastases. Patient elected to delay the next line of therapy and passed away on day 206 (week 29), likely secondary to disease progression.

## Case presentation #2

A 39-year-old women with refractory and metastatic SS was treated with fludarabine–cyclophosphamide lymphodepletion (Table 1) followed by adoptive transfer of NY-ESO-1^c259^ T-cells. She has a history of an 8 cm left thigh SS, initially treated with pre-operative radiation, wide surgical resection with negative margins, and 4 cycles of adjuvant doxorubicin/ifosfamide chemotherapy. She developed metastatic disease to lung, and local thigh recurrence 10 and 14 months, respectively, after completion of primary therapy. Subsequent oncologic treatments included lung wedge resection, palliative radiation to the left thigh recurrence to 50.4 Gy, and pazopanib. Her tumor also had low NY-ESO-1 expression, as defined previously.

The patient’s post T-cell infusion course was complicated by nausea, vomiting, pancytopenia, and grade 2 CRS as evidenced by hypotension, headache, low grade fever, rash, and elevated CRP and ferritin. The patient received IV fluids and tocilizumab (8 mg/kg) on Day 7. The patient’s counts recovered, symptoms of CRS resolved, and she was discharged day 12. Unfortunately, the patient was re-admitted day 15 with fever, grade 3 morbilliform rash, severe diarrhea, hypotension not responsive to IV fluids, elevated ferritin, and mild transaminitis, but normal CRP. The patient was felt to have recurrent CRS and a second dose of tocilizumab (8 mg/kg) was given on day 17. A skin punch biopsy was consistent with acute spongiotic dermatitis with dyskeratotic keratinocytes. The rash was felt to be a drug reaction to Bactrim. Additionally, reactivation of HHV-6 viral was identified, without any neurological symptoms, which may have contributed to the transaminitis. HHV-6 viral load then became undetectable. By Day 24 the patient showed improvement and was discharged to home. The patients’ course was further complicated by severe pain and swelling in the left inguinal/hip area, at the site of her prior palliative radiation, attributed to radiation recall phenomenon; improved with a short course of corticosteroids. CMV reactivation, on day 32, treated with course of valganciclovir with resolution. Furthermore, the patient developed a bacteremia and a supra-infected necrotic left pelvic tumor, cultures positive for ESBL *E. coli*, treated with 6 weeks of IV ertapenem, and then oral suppressive anti-biotics with Augmentin. Restaging evaluation at one, two, and 3 months show stable lung nodules and pelvic metastasis.

At the month 4 follow-up visit, the subject presented with bilateral foot numbness, left foot drop, unsteady gait, and pain in the left thigh. An outpatient work-up was initiated with neurology consultation. Two weeks later the patient’s symptoms had significantly worsened with BLE weakness, inability to walk, numbness bilaterally in the hands, legs, and feet, and worsening burning pain in the left anterior thigh, which radiated down to the left knee and left lower leg. Physical examination revealed loss of DTRs in the BLEs, bowel incontinence, BLE weakness right > left, inability to ambulate, and decrease sensation to light touch, pinprick, and vibration in the finger tips and left lower extremity. No myoclonus, tremor, or fasciculations. The patient was alert and oriented, upper extremity strength intact, cranial nerve examination unremarkable.

Given these worsening neurological symptoms of peripheral sensory and motor neurotherapy the patient was admitted on day 128 for further work-up. An MRI of the C/T/L spine was negative for spinal cord compression, no transvers myelitis, but showed non-specific enhancement of the conus and roots of the cauda equina. A lumbar puncture (LP) revealed no malignant cells, low cell count, no bacteria, elevated protein 134.2 mg/dl, and negative viral studies for West Nile, Enterovirus, HSV, and negative for *B. Burgdorferi*. Serum CMV, EBV, and VZV DNA undetectable, as well. The patient did not have diarrhea, and a prior stool culture showed no campylobacter. Autoimmune laboratory work-up included trace anti-nuclear antibody, CRP 5.0 mg/L, ESR 59 mm/h, and SPEP with immunofixation showed a faint monocloncal gammopathy with free lambda paraprotein. The nerve conduction studies showed evidence of a non-length dependent demyelinating sensorimotor polyneuropathy consistent with acute inflammatory demyelinating polyradiculoneuropathy (AIDP), a variant of Guillain-Barré Syndrome (GBS).

The patient was subsequently started on 0.4 g/kg/day of intravenous immunoglobulin (IVIG) over 5 days. The day after completion of IVIG the patient noticed improved strength. She was discharged to rehabilitation facility, then to home after approximately 2 weeks. At the 6-month follow-up visit, the patients’ strength and sensory neuropathy were continuing to improve though she still had some weakness in the left lower leg and was using a walker to ambulate. At the 8-month follow-up visit, the patient’s pain was well controlled, sensory neuropathy fully resolved, and strength returned to normal, able to ambulate without assistance.

Unfortunately, the patient developed disease progression, on the 6-month (day 163) follow-up surveillance scans, as evidenced by mild enlargement of the pelvic metastasis. The patient had not sufficiently recovered from her AIDP to be a candidate for systemic chemotherapy, and a period of short-term observation was recommended. At the 8-month (day 240) follow-up surveillance scans showed new pulmonary nodules, and significant enlargement of her pelvic tumor with intra-tumoral hemorrhage. The patient was taken off protocol and received systemic chemotherapy with trabectedin for one cycle with disease progression. The patient died on day 278 secondary to disease progression.

## Discussion

NY-ESO-1^c259^ T cells are generally well tolerated, with the most common treatment-related adverse events being hematologic toxicities from preparative chemotherapy regimens. Cytokine release syndrome (CRS), all grades and grade 3+, occurred in 42 and 17% of patients, respectively [13]. Unlike CD19-specific chimeric antigen receptor (CAR) T-cell therapy which can cause serious neurological adverse events like seizure, cerebral edema, or encephalopathy, the administration of NY-ESO-1^c259^ T cells has not been linked with such toxicities [13].

To the best of our knowledge, these are the first two reports of polyneuropathy development post adoptive transfer of NY-ESO-1^c259^ T lymphocytes. Patient 2 had a definitive diagnosis of AIDP based on high CSF protein and nerve conduction studies, but patient 1 based on the NCS has a diagnosis suggestive of a mild, distal, axonal polyneuropathy. Although both patients presented with several classic symptoms of polyneuropathy (progressive symmetric muscle weakness, absent/depressed DTRs, impaired sensation, ataxic gait, and mild dysautonomia), patients may present with atypical symptoms/laboratory results, as seen with the first case. Patient 1 had initial patellar/achilles DTR of 0 on first presentation to ER (day 42) that improved to brisk + 2 DTRs of the lower extremities by day 49, the day before starting IVIG, and had no albuminocytologic dissociation in CSF, although this may be confounded by the fact that the patient had already received one dose of IVIG. Albuminocytologic dissociation was not appreciated in patient one; however, normal CSF protein does not exclude the diagnosis of GBS/AIDP. Also, the NCS showed very mild changes, thus supporting a possible diagnosis of a mild distal motor axonal polyneuropathy. Therefore, it is important to remain vigilant in recognizing both typical and atypical neurological symptoms that can happen at any time after the T-cell infusion (as seen with the different time to symptom onset) for prompt workup and diagnosis. Other etiologies including critical illness polyneuropathy, infectious etiologies, aseptic meningitis, brain metastases, leptomeningeal disease, spinal cord compression and transverse myelitis were ruled out.

Of note, GBS/AIDP has been noted as a rare complication of allogenic bone-marrow transplantation (BMT), which can develop 2 days to 15 months after BMT. The pathogenesis is unclear, with proposed mechanisms including infections, drug toxicity, and graft-versus host disease [14, 15]. Another possible etiology of GBS/AIDP includes immune reconstitution inflammatory syndrome (IRIS), as seen with HIV patients quickly after starting therapy or stem cell transplant patients when immunosuppression is reduced [16]. This phenomena of immune upregulation may also explain the rare toxicity of GBS/AIDP seen with checkpoint inhibitors used in melanoma [17]. Melanocytes express highly immunogenic gangliosides that are also expressed on Schwann cells in the peripheral nervous system; thus, antibody formation against melanoma cells may also lead to immune-mediated neurotoxicities, including GBS/AIDP [18]. Of note, normal Schwann cells and neurons, under normal physiological conditions, do not express HLA, which is required for on and off-target recognition of the study drug [19]. It remains possible that the study drug influenced subsequent development of GBS through an indirect modulation of an immune response to infection.

GBS has been associated with CMV [20], and reported with HHV6 infection [21]. Though reactivation of CMV and HHV6 were noted in patient #2 within the first 30 days of her T cell infusion, the patient had undetectable CMV and HHV6 DNA at the time of the GBS/AIDP diagnosis. Thus, it is plausible that GBS is an untoward adverse event of adoptive transfer of T-lymphocytes.

## Conclusion

In this report, we described two patients with metastatic SS status post adoptive T-cell therapy with NY-ESO-1^c259^ T-cells who subsequently developed GBS/AIDP. Prompt recognition of symptoms and early consultation with neurology specialists is essential to rule out alternative etiologies and initiate treatment in this rapidly debilitating disease state. Given the expanding applications of immunotherapy in cancer management, clinicians should stay vigilant against the potential development of unusual but life-threatening immune-mediated toxicities.

## Data Availability

Identifying patient information must remain confidential; however, additional data may be available upon reasonable request at the discretion of the corresponding author.

## Abbreviations

AIDP: Acute inflammatory demyelinating polyneuropathy
BLE: Bilateral lower extremities
BMT: Bone marrow transplant
CAR T-cell therapy: Chimeric antigen receptor (CAR) T-cell therapy
CRS: Cytokine release syndrome
CSF: cerebrospinal fluid
DFCI: Dana-Farber Cancer Institute
DTR: Deep tendon reflexes
EMG: Electromyography
GBS: Guillain-Barre syndrome
IVIG: Intravenous immunoglobulin
LP: Lumbar puncture
NCS: Nerve conduction studies
NY-ESO-1: New York – esophageal squamous cell carcinoma-1
SS: Synovial sarcoma
STS: Soft-tissue sarcoma
